# On the Operative Surgery of Malignant Disease

**Published:** 1887-12

**Authors:** 


*On the Operative Surgery of Malignant Disease.* By HENRY T. BUTLIN, F.R.C.S. Pp. 408. London: J. & A. CHURCHILL. 1887.

The author has done good service to his profession by publishing this work. It is a careful, almost laborious, investigation of the results of operations for malignant disease, and an honest and judicial series of deductions as to the propriety of these operations, and the best time and best manner of performing them. In its fullest sense the work is one of prognosis; enabling the surgeon to state to a patient with malignant disease what is his expectation of life after submitting to operation. In this sense the book stands alone; there is nothing to compete with it; and if the book had contained nothing else, the operating surgeon could not have failed to have recognised its value. But it contains much more. Not only are the operations described, and usually well described, but the whole of the circumstances antecedent thereto—including diagnosis; and subsequent thereto—including the after-history of the patient, which we are so liable to consider as ceasing when he has left with the wound healed,—are entered into with a care and minuteness which is worthy of all praise.

It is essential that on a certain date an author must cease writing his book, and hand it over to the printer. No book can be up to the date of the hour at which it is placed in the hands of the public. It would be unfair to the author to point out that his statistics in some of the operations must already be modified; this he will himself have already seen, and no doubt have noted, in view of a new edition.

Altogether we look upon the work, not only as being valuable from the material it contains, but as being specially commendable for the conspicuous power and honesty with which the material has been handled for the advancement of surgery.

